# Facial Nerve Paralysis Revealing Adenosquamous Carcinoma of the Parotid Gland: A Case Report

**DOI:** 10.7759/cureus.108266

**Published:** 2026-05-04

**Authors:** Nourelhouda Remok, Jalal El Kidari, Elmahdi Choukri, Bouchra Dahmani, Siham Alaoui Rachidi

**Affiliations:** 1 Faculty of Medicine and Pharmacy, Abdelmalek Essaâdi University, Mohammed VI University Hospital, Tangier, MAR; 2 Department of Radiology, CHU Mohammed VI Tangier, Tangier, MAR

**Keywords:** adenosquamous carcinoma, facial nerve paralysis, parotid gland tumor, peripheral facial palsy, pulmonary metastases

## Abstract

Adenosquamous carcinoma (ASC) of the salivary glands is a rare and aggressive malignancy. We report the case of a patient who presented to the otorhinolaryngology department with a two-month history of left parotid swelling associated with peripheral facial nerve paralysis. Imaging revealed an ill-defined, expansive lesion of the left parotid gland with intracranial extension and heterogeneous enhancement following intravenous contrast administration, along with concomitant pulmonary metastases. A surgical biopsy confirmed the diagnosis of primary ASC of the salivary gland. The patient was treated with chemotherapy, with follow-up demonstrating a reduction in the size of the primary tumor and regression of pulmonary metastases. This report highlights the aggressive behavior of this rare entity and underscores the essential role of imaging in its diagnosis and management.

## Introduction

Adenosquamous carcinoma (ASC) is a rare and highly aggressive malignancy of the head and neck region, predominantly arising from mucosal surfaces of the upper aerodigestive tract, with only occasional involvement of the salivary glands [1]. Histologically, ASC is defined by its biphasic composition, combining both squamous and glandular (adenocarcinomatous) differentiation [2]. According to the World Health Organization classification, it is considered a variant of squamous cell carcinoma and accounts for fewer than 1% of all salivary gland malignancies [2].

Among the salivary glands, the parotid gland is most commonly affected. The disease typically occurs in older adults and shows a slight male predominance. Clinically, patients often present with a rapidly enlarging parotid mass, frequently associated with pain or facial nerve palsy. The latter is considered a red flag for malignancy, as it reflects tumor invasion of the facial nerve, a feature associated with advanced disease. ASC is characterized by an aggressive clinical course, with a high propensity for early metastasis and an overall poor prognosis, particularly in advanced stages [3]. Imaging plays a pivotal role in the evaluation of parotid malignancies. Contrast-enhanced CT is valuable for assessing tumor extent, osseous involvement, and cervical lymphadenopathy. MRI, with its superior soft-tissue resolution, is essential for evaluating perineural spread, defined as tumor extension along nerve sheaths, as well as deep lobe and skull base involvement. These imaging features are critical for accurate staging and therapeutic planning [4].

Management of ASC typically requires a multidisciplinary approach. Surgical resection remains the cornerstone of treatment when feasible, often combined with adjuvant radiotherapy and/or systemic therapy in advanced cases. Despite these interventions, prognosis remains poor due to the tumor’s aggressive behavior and high rates of recurrence and metastasis. Given the rarity of this entity, there is no standardized treatment protocol, and management strategies are generally individualized [5,6]. We report a case involving a 60-year-old male patient, characterized by unusual intracranial extension and a notable favorable response to chemotherapy, underscoring the importance of imaging in diagnosis, staging, and treatment assessment.

## Case presentation

We report the case of a 60-year-old male, a former chronic smoker, who developed a left parotid swelling over two months, associated with ipsilateral peripheral facial nerve paralysis. On physical examination, the patient was hemodynamically and respiratorily stable and afebrile. A firm, tender left parotid mass measuring approximately 4 cm in diameter was noted, with no overlying inflammatory changes. The clinical presentation was associated with generalized fatigue.

**Figure 1:**
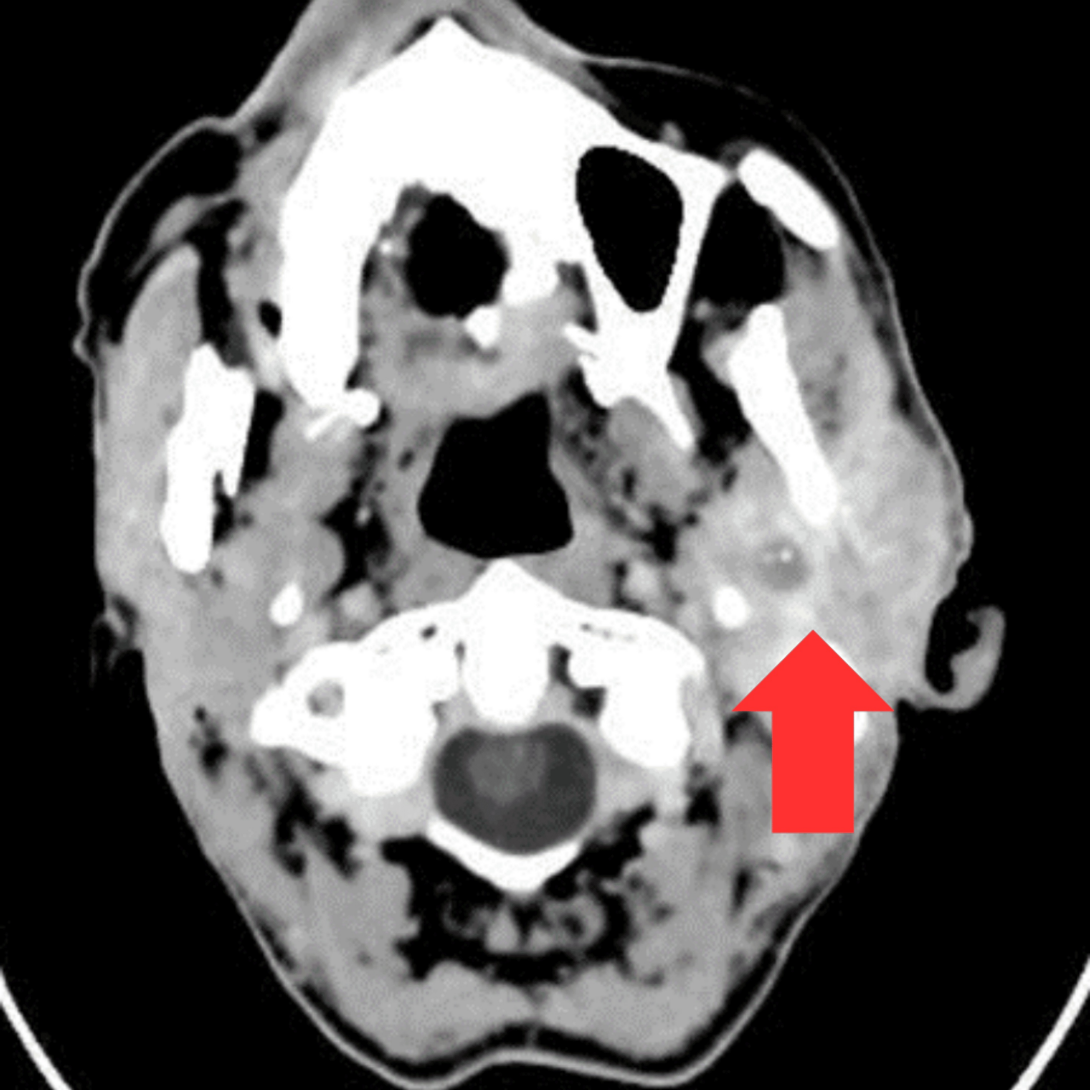
Brain CT post-contrast in mediastinum window showing a voluminous, ill-defined, heterogeneous soft-tissue mass of the left parotid gland, involving both the superficial and deep lobes and resulting in glandular enlargement CT: computed tomography

**Figure 2:**
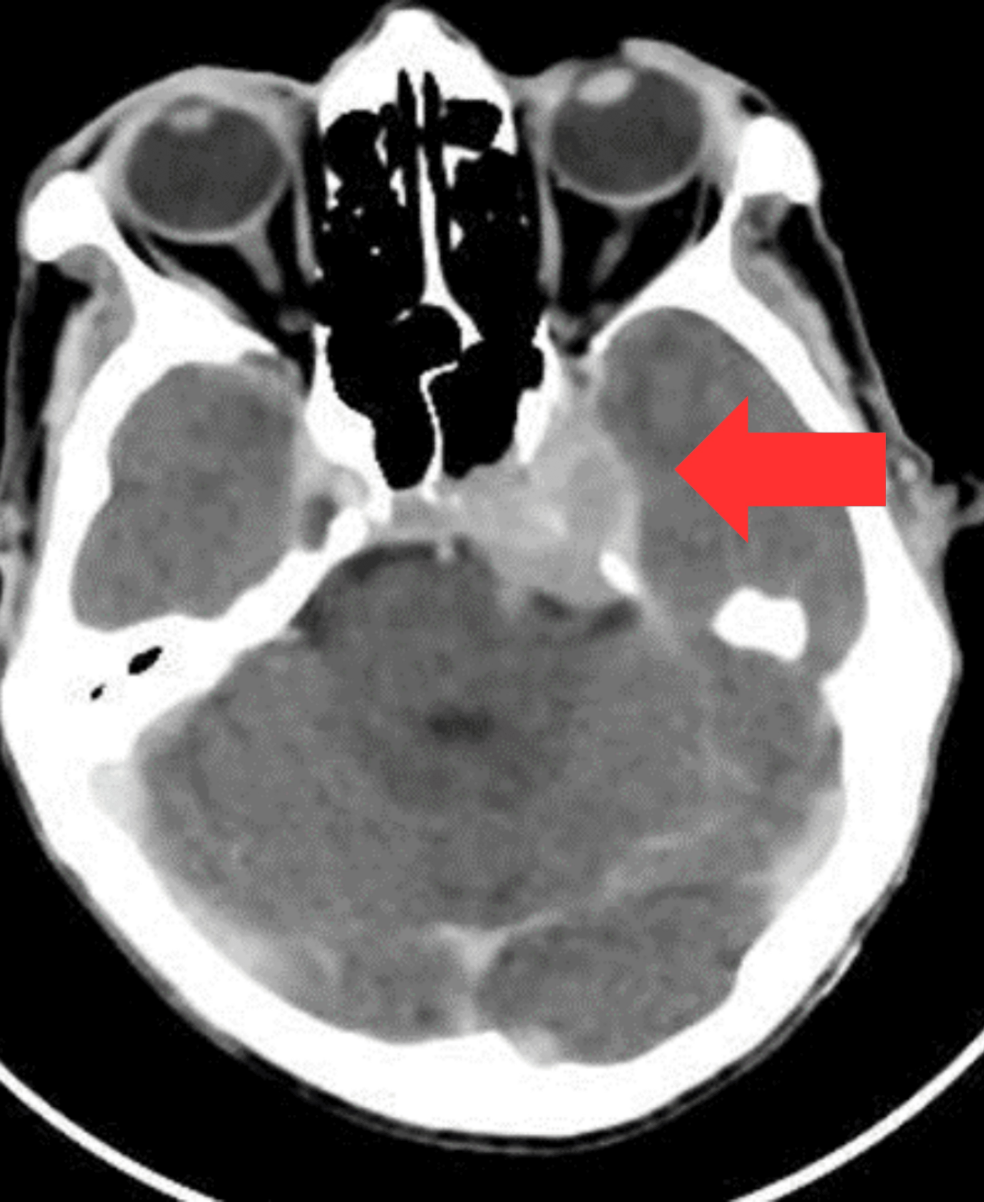
Brain CT post-contrast in mediastinum window demonstrating intracranial extension of the lesion, forming an extra-axial mass in the petroclival region, exerting mass effect on the temporal lobe and obliterating the prepontine cistern CT: computed tomography

Initial imaging with CT of the brain was followed by whole-body CT staging. Brain CT demonstrated a voluminous, ill-defined mass involving both the superficial and deep lobes of the left parotid gland, resulting in glandular enlargement. The lesion showed intracranial extension with mass effect on the adjacent temporal lobe (Figures 1, 2).

**Figure 3:**
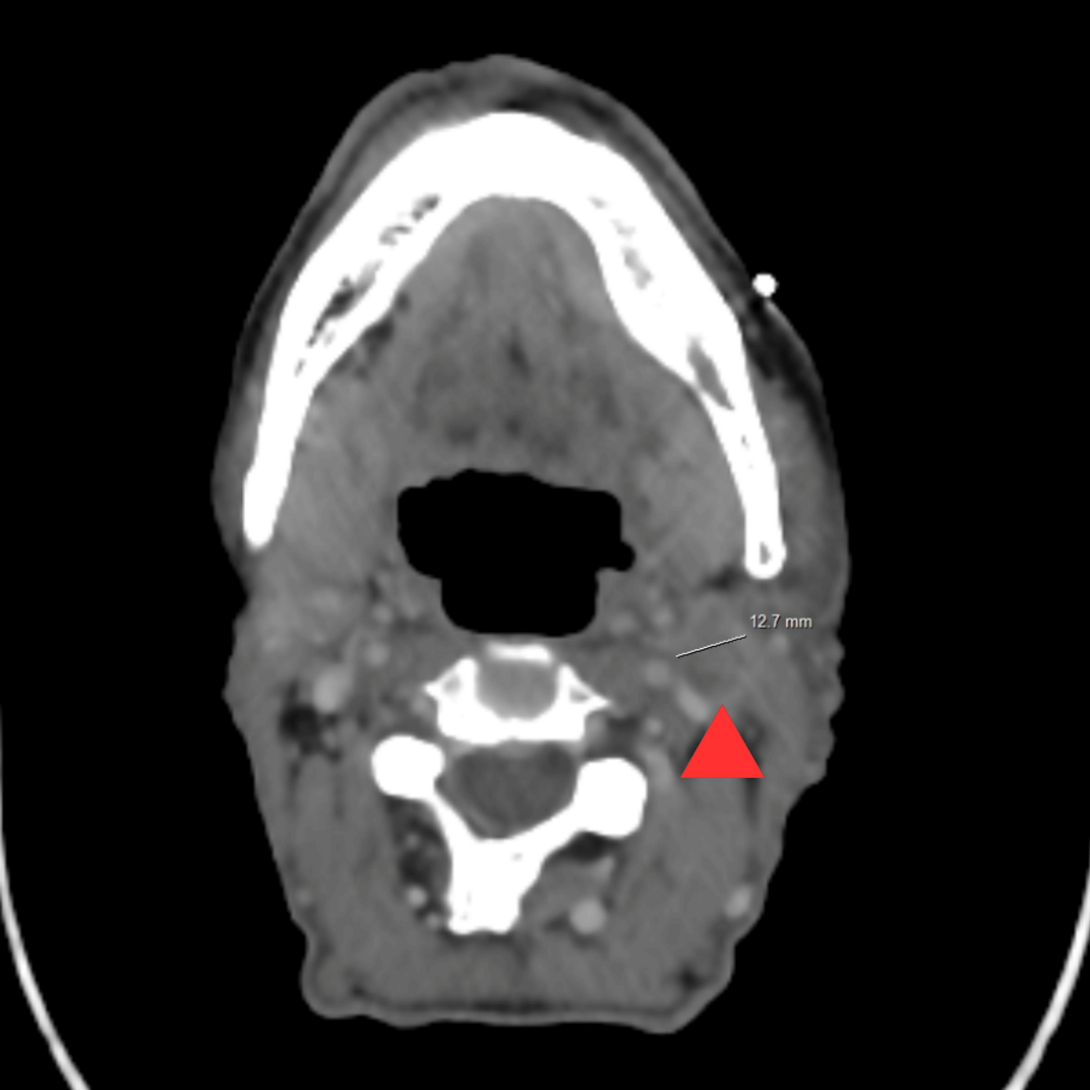
Post-contrast cervical CT in the mediastinal window demonstrates a left cervical lymph node with central necrosis, measuring 13 mm in short axis CT: computed tomography

**Figure 4:**
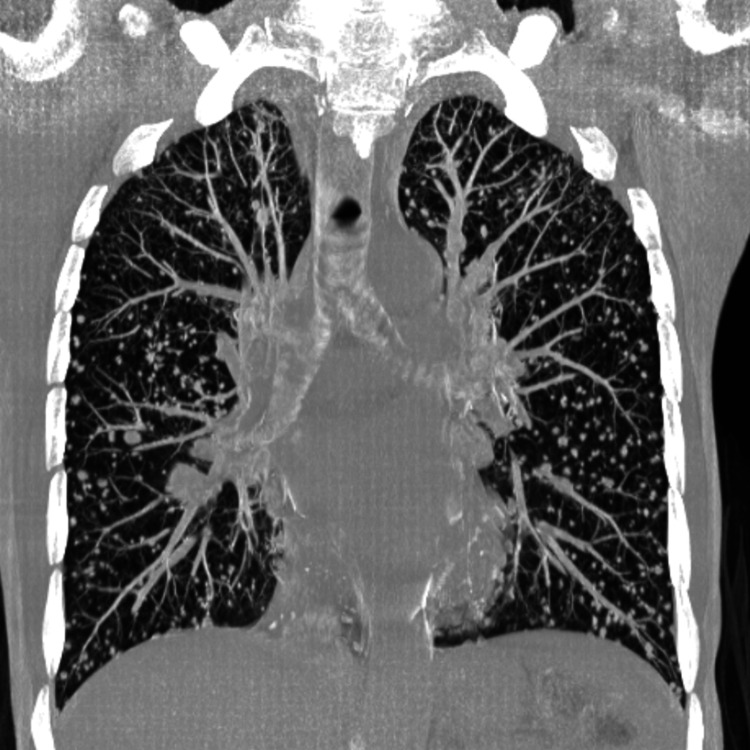
Thoracic CT in the lung window (coronal plane, MIP reconstruction) shows multiple pulmonary nodules and micronodules of varying shape and size, consistent with metastatic disease CT: computed tomography; MIP: maximum intensity projection

Whole-body CT revealed left superior jugulocarotid cervical lymphadenopathy as well as multiple pulmonary metastases (Figures 3, 4). 

Following biopsy, which was performed the day after imaging, histopathological results were available one week later. Immunohistochemical analysis demonstrated positivity for markers supporting both squamous and glandular differentiation, including p40 and cytokeratin 7, confirming the diagnosis of ASC of the parotid gland. The case was subsequently discussed in a multidisciplinary tumor board meeting, and systemic chemotherapy with cisplatin and capecitabine was selected based on institutional practice for advanced, unresectable, and metastatic salivary gland malignancies, given the absence of standardized treatment protocols for this rare entity.

Systemic chemotherapy was initiated one month after the initial imaging workup. A follow-up CT performed eight months later demonstrated a significant therapeutic response, with approximately a 45% reduction in the size of the primary left parotid mass according to RECIST 1.1 (Response Evaluation Criteria in Solid Tumours) criteria, along with regression of intracranial extension and a decrease in pulmonary metastatic lesions. Clinically, the patient’s general condition improved; however, ipsilateral facial nerve paralysis persisted without significant recovery at follow-up.

## Discussion

ASC of the salivary glands is a rare and aggressive malignancy with limited published data, which makes its clinical management challenging [5]. In the present case, the rapid evolution of a parotid swelling associated with peripheral facial nerve paralysis reflects the aggressive clinical behavior of this tumor and is consistent with an advanced disease presentation described in the literature. In this context, imaging was particularly valuable in confirming the aggressive behavior of the disease and in establishing the extent of dissemination. The overall radiological profile supported a high-grade salivary malignancy with early systemic spread, which is consistent with the known aggressive nature of ASC [5]. These findings were essential for accurate staging and directly influenced the decision to pursue systemic rather than locoregional therapy.

Histopathological analysis remains the gold standard for diagnosis, as imaging findings are non-specific and may overlap with other aggressive salivary malignancies [1]. In this case, a core needle biopsy confirmed ASC, enabling appropriate therapeutic planning. Given the advanced stage at presentation with systemic involvement, therapeutic options were limited, and management had to be individualized. In such cases, systemic therapy is generally considered the most appropriate approach, as curative surgical resection is not feasible in metastatic disease. The decision to initiate platinum-based chemotherapy aligns with reported approaches for high-grade salivary gland malignancies, where systemic regimens are often extrapolated from squamous cell carcinoma protocols due to the lack of standardized treatment guidelines [5,6]. The partial radiological response observed suggests that some tumors may exhibit chemosensitivity, although outcomes remain guarded overall.

This report highlights the importance of recognizing facial nerve paralysis as a red flag for malignancy, the key role of imaging in staging and follow-up, and the need for individualized multidisciplinary management in rare and aggressive salivary gland tumors.

## Conclusions

ASC of the parotid gland is a highly aggressive malignancy that frequently presents at an advanced stage. This report highlights the importance of recognizing facial nerve paralysis as a key clinical indicator of malignant involvement. Comprehensive imaging combined with histopathological confirmation is essential for accurate staging and appropriate management. In the absence of standardized treatment strategies, therapeutic decisions must be individualized, particularly in the setting of metastatic disease. The observed response to cisplatin-based chemotherapy suggests that selected patients may benefit from systemic treatment, although overall prognosis remains unfavorable. In this case, the follow-up period was limited, and therefore, long-term survival outcomes and durability of response could not be assessed. Early diagnosis, multidisciplinary management, and close follow-up remain essential to optimize patient care and improve outcomes.
